# New Possible Ways to Use Exosomes in Diagnostics and Therapy via JAK/STAT Pathways

**DOI:** 10.3390/pharmaceutics15071904

**Published:** 2023-07-07

**Authors:** Gréta Gombos, Nikolett Németh, Ondrej Pös, Jakub Styk, Gergely Buglyó, Tomas Szemes, Ludovit Danihel, Bálint Nagy, István Balogh, Beáta Soltész

**Affiliations:** 1 Department of Human Genetics, Faculty of Medicine, University of Debrecen, Egyetem Tér 1, H-4032 Debrecen, Hungary; gombos.greta@med.unideb.hu (G.G.); nemeth.nikolett@med.unideb.hu (N.N.); gbuglyo@hotmail.com (G.B.); nagy.balint@med.unideb.hu (B.N.); balogh@med.unideb.hu (I.B.); 2 Comenius University Science Park, 841 04 Bratislava, Slovakia; ondrej.pos@uniba.sk (O.P.); jakub.styk@gmail.com (J.S.); tomas.szemes@uniba.sk (T.S.); 3 Geneton Ltd., 841 04 Bratislava, Slovakia; 4 Institute of Medical Biology, Genetics and Clinical Genetics, Faculty of Medicine, Comenius University, 811 08 Bratislava, Slovakia; 5 Department of Molecular Biology, Faculty of Natural Sciences, Comenius University, 841 01 Bratislava, Slovakia; 6 3rd Surgical Clinic, Faculty of Medicine, Comenius University and Merciful Brothers University Hospital, 811 08 Bratislava, Slovakia; l.danihel@gmail.com; 7 Division of Clinical Genetics, Department of Laboratory Medicine, Faculty of Medicine, University of Debrecen, H-4032 Debrecen, Hungary

**Keywords:** exosomes, JAK/STAT pathways, TRIM, tumor-derived exosomes, cancer

## Abstract

Exosomes have the potential to be the future of personalized diagnostics and therapy. They are nano-sized particles between 30 and 100 nm flowing in the extracellular milieu, where they mediate cell–cell communication and participate in immune system regulation. Tumor-derived exosomes (TDEs) secreted from different types of cancer cells are the key regulators of the tumor microenvironment. With their immune suppressive cargo, TDEs prevent the antitumor immune response, leading to reduced effectiveness of cancer treatment by promoting a pro-tumorigenic microenvironment. Involved signaling pathways take part in the regulation of tumor proliferation, differentiation, apoptosis, and angiogenesis. Signal transducers and activators of transcription factors (STATs) and Janus kinase (JAK) signaling pathways are crucial in malignancies and autoimmune diseases alike, and their potential to be manipulated is currently the focus of interest. In this review, we aim to discuss exosomes, TDEs, and the JAK/STAT pathways, along with mediators like interleukins, tripartite motif proteins, and interferons.

## 1. Introduction

Exosomes are nano-sized extracellular vesicles carrying donor cell-derived markers such as nucleic acids, proteins (including receptors and enzymes), and lipids that may provide practical implications relying on the fact that most cell types secrete exosomes easily accessible by non-invasive liquid biopsy-based approaches [1]. These vesicles can mediate cancer progression and metastasis or reduce the cytotoxic effect of antitumor therapy [2]. On the other hand, exosomes can be engineered and delivered to recipient cells to modify the tumor microenvironment and enhance the effectiveness of cancer therapy in patients [3]. Nowadays JAK/STAT is also procured in the cancer-associated pathways, thus its modulation via exosomes is gaining attention in potential therapeutic applications.

## 2. Exosome Biogenesis

**Figure 1:**
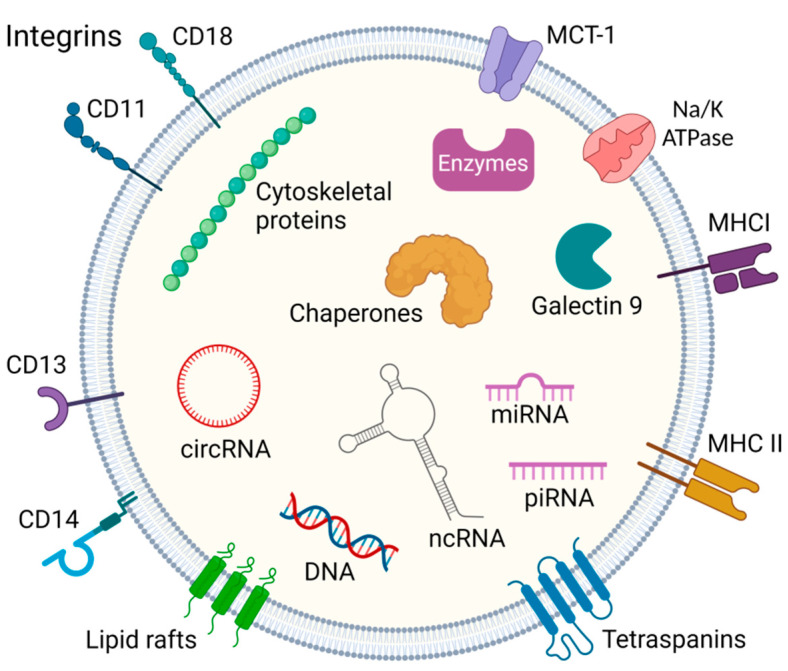
General constituents of the exosome. Exosomes contain proteins, endosome-specific proteins, cytoskeletal proteins, chaperones and enzymes, and various nucleic acids such as miRNA, PIWI-interacting RNA, circular RNA, and other non-coding RNA. Their membranes also contain abundant molecules such as integrins, tetraspanins, MHC I/II- major histocompatibility complex, MCT-1-Monocarboxylate transporter 1, and clusters of differentiation (CD) markers [4,5,8,9,10,11,12,13,14]. Created with BioRender.com.

Exosomes are unique vesicles carrying numerous biomarkers, that may provide potential for personalized diagnostics and therapy. They are nano-sized particles between 30 and 100 nm in the extracellular milieu, mediating cell–cell communication and participating in the immune system. The exosome biogenesis starts with the intrusion of the plasma membrane, leading to an early endosomal shape in which low-density vesicles are very close to the surface of the inner membrane [4,5]. A particular collection of cytosolic factors on the endosomal membrane manages the invagination of the endosomal lipid bilayer towards their luminal space, leading to the creation of intraluminal vesicles. Such a process, called early endosome maturation, results in the formation of multivesicular bodies [6] that can fuse with cell membranes via an adenosine triphosphate (ATP)-dependent process, thus releasing exosomes into the neighboring extracellular space [7]. In terms of structure, exosomes are encompassed by a phospholipid bilayer containing integral membrane proteins and lipid rafts. Some transmembrane proteins are found in a glycosylated form, which allows other proteins to bind using their carbohydrate side chains. The role of the outer bilayer is to protect the molecules transported inside, while the content is regulated by the endosomal sorting complex required for transport [4,5]. Among the transported molecules, annexins, flotillins, Rab proteins, CD8, CD13, CD14, CD9, CD63, CD81, and many other proteins characteristic of the donor cell may be present. In addition to proteins, exosomes may also contain different nucleic acids, e.g., DNA, mitochondrial DNA (mtDNA), RNAs, such as microRNA (miRNA), long non-coding RNA (lncRNA), and piwi-interacting RNA (piRNA) (Figure 1) [5,8,9,10,11,12,13,14,15].

### 2.1. Exosomal Cargo Uptake via JAK/STAT Dependent Pathway

Recipient cells may incorporate exosomal cargo through membrane diffusion or via a receptor-mediated pathway employing tripartite motif (TRIM) proteins [5,8,9,10,11,12,13,14]. The TRIM protein family is known for its regulatory roles in transcription, apoptosis, and cellular growth, and their effect on the Janus kinases (JAKs) and signal transducer and the activator of transcription proteins (STATs) signaling pathways has also been described [16]. Some TRIMs are frequently found in specific tumors. For example, TRIM44 has modulatory effects on tumor progression and is a potential indicator of unfavorable prognosis in epithelial ovarian cancer [17]. TRIM44 expression is positively regulated via ovarian cancer-derived exosomal circRNA nuclear factor I X (circNFIX) [18]. The JAK/STAT pathway is regulated by ovarian cancer-derived exosomal circNFIX in human umbilical vein endothelial cells through the miR518a-3p/TRIM44 axis (Table 1). circNFIX and TRIM44 correlate with the tumor stage as defined by the International Federation of Gynecology and Obstetrics (FIGO) and with the size of ovarian tumors [18]. The effect of TRIM44 was shown to be modified in a microRNA-dependent manner. The miR-34a-5p may directly target TRIM44, thus inhibiting its activity and leading to suppression of ovarian cancer angiogenesis and malignant behavior [18]. Also, TRIM66 may advance the expression of the JAK/STAT pathways in prostate carcinoma. TRIM66 is part of the signaling axis composed of TRIM66-STAT2-IL-2 molecules, in which TRIM66 favorably regulates the expression of STAT2 and IL-2. It was found that overexpression of either STAT2 or IL-2 almost completely abolished the inhibitory effects of TRIM66 deficiency on cell proliferation, migration, and invasion. This demonstrates the predominance of STAT2-IL-2 in mediating the oncogenic properties of TRIM66 [19].

**Table 1:** The role of TDE-derived RNAs, DNA, and proteins in onco-immunology and JAK/STAT pathways in various cancer types.

Cancer	Source	Biomarker	Function	References
Ovarian cancer	RNA	circNFIX, miR-518a-3p	Correlates with FIGO and tumor size progression	[18]
Lung cancer	RNA	miR-21, miR-29a	Connects to TLR-8 pro-inflammatory cytokines, metastasis, tumor proliferation	[2]
Multiple myeloma	RNA	miR-146a	Activates JAK-STAT pathways via Notch pathway	[3,20,21,22]
Gastric cancer	RNA	miR-3184-5p	Suppresses cell proliferation, migration, and invasion downregulating inter alia the expression of p-STAT3	[23,24,25]
Prostate cancer	RNA	miR-187	Suppresses malignancy via JAK3/STAT3 pathway	[26]
	RNA	miR-222-3p	Overexpression promotes cell proliferation	[27]
Renal cell carcinoma	RNA	lncARSR	inducing macrophage polarization by activating STAT3	[28]
NSCLC	DNA	mutated *KRAS*	Switch naive CD4+ T cells to Treg-like cells	[29]
Pancreatic cancer	Protein	KRAS	Promotes macrophage polarization (M2) via STAT3	[30]
Breast cancer	Protein	gp130	Increasing the levels of pro-tumorigenic cytokines via STAT3	[31]
Lung cancer	Protein	hsp72	STAT3-dependent immunosuppressive function	[2]

### 2.2. Biological Roles of Tumor-Derived Exosomes in Tumor Microenvironment

**Figure 2:**
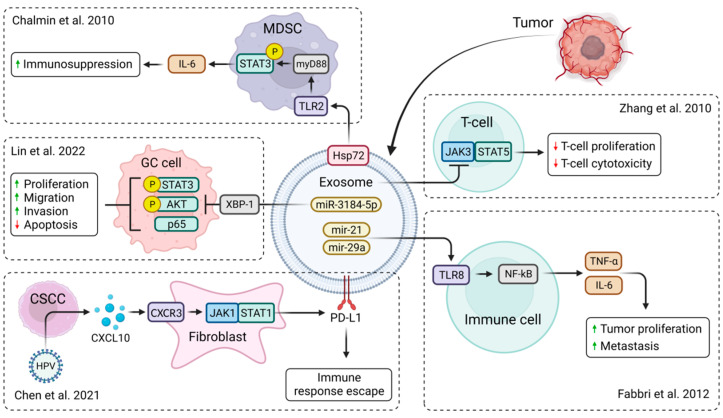
Biological functions of TDEs in tumor microenvironment. Membrane-associated Hsp72 from TDEs mediates STAT3-dependent immunosuppressive function of myeloid-derived suppressor cells (MDSC) [54]. miR-3184-5p expression was downregulated in gastric cancer (GC) patients, resulting in increased cell proliferation, migration, and invasion and reduced cell apoptosis [25]. CXCL10 produced by HPV-positive cervical squamous cell carcinoma (CSCC) stimulates exosomal PDL1 expression by fibroblasts via CXCR3 and JAK-STAT pathways, forming an immune escape response pathway that leads to tumor emergence [53]. TDEs from renal cancer cells suppressed the expression of JAK3 and p-STAT5 in T lymphocytes, reducing T-cell proliferation and cytotoxic effects [35]. Membrane-associated Hsp72 from TDEs mediates STAT3-dependent immunosuppressive functions [2]. Created with BioRender.com.

It has been shown that exosomes may be derived from different types of cancer cells. Such tumor-derived exosomes (TDEs) can play an important role in cancer progression and metastasis by decreasing cytotoxicity and promoting a pro-tumorigenic microenvironment [32]. Their immune suppressive cargo prevents the antitumor immune response, thus reducing the effectiveness of cancer treatment (Figure 2). However, TDEs act as a double-edged sword, as they may also present tumor antigens promoting anti-tumor immune responses [33].

This is of great therapeutic interest, as exosomes can be engineered to express receptors enhancing the specificity of their target cell binding, increasing their efficacy, and reducing deleterious off-target effects [34]. Moreover, attempts to anchor interleukins (IL), e.g., IL-12, to the exosome membrane with glycosylphosphatidylinositol anchor technology showed great results in terms of inducing T-cell proliferation (Figure 2). Higher IFN-γ release and higher cytotoxic effects were also observed in comparison to untreated exosomes [35]. A study by Zhang et al., revealed that renal cell carcinoma-derived exosomes could deliver lncRNAs (Figure 1), namely, lncARSR, inducing macrophage polarization by activating the STAT3 signaling pathway that causes changes in cytokine secretion and phagocytosis, thereby promoting tumor development [28]. Other studies found that renal TDEs also downregulate JAK3 expression and STAT5 phosphorylation at high doses in T lymphocytes with no effect on JAK2. Similar to TDEs, renal cancer TDE-anchored IL-12 (EXO/IL-12) also provides a much smaller reduction in p-STAT5 expression but has the same inhibitory effect on JAK3, but not on JAK2. The induction of T lymphocyte cytotoxicity enhanced by EXO/IL-12 partly depends on the upregulation of p-STAT5 expression. In conclusion, EXO/IL-12 may phosphorylate STAT5 via an alternative approach to the JAK2/STAT5 pathway when TDEs suppress the JAK3/STAT5 pathway in T lymphocytes [35,36]. LncRNAs in TDEs associated with breast cancer brain metastasis boost JAK2 activity that activates STAT3. This leads to macrophage activation and recruitment, promoting breast cancer brain metastasis as exosomes penetrate the blood-brain barrier [37].

Studies have shown that the development of cancer cachexia correlates with exosome concentration [38]. C26 cell-secreted exosomes can reduce the C2C12 myotube diameter in vitro and even reduce tibialis anterior muscle weight along with mouse grip strength in vivo [39]. The expression levels of STAT3 and STAT3 in C26 cells and the exosome release from C26 cells were decreased by siRNA [40]. In C26 cells, the overexpression of the STAT3 upregulates exosome biogenesis in sync with the level of exosomal markers. These results foreshadow that the STAT3 expression is linked with the exosome biogenesis in the C26 tumor cell line. Examination of the exosomes with electron microscopy showed that the TDEs in this case have a cup-shaped morphology with a diameter of about 50.7 nm [38,41]. In comparison to the control cell group, the culture medium of the C26 knockdown mock cells and the C26 overexpressed mock groups indicated a significant decrease in the diameter of the myotubes [39]. On the other hand, the culture medium of the C26-STAT3 knockdown cells only induced a mild subside in the diameter; in contrast, the culture medium of the C26-STAT3-overexpressed cells showed an enhanced decrease. In C26 cells, the knock-out STAT3 significantly reduces glycolysis [38,42]. Inhibiting the STAT3 phosphorylation henceforward reduces the transcriptional levels of HIF-1α, HK2, and serine hydroxymethyltransferase 2 along with pyruvate kinase M2 (PKM2) activation, which affects the synaptosome-associated protein 23 (SNAP23) activation, thus regulating exosome biogenesis [43]. IL-6 has been reported to play a pivotal role in the induction of muscle atrophy and lipolysis. It is also known that IL-6 binds to its receptors forms a complex that activates the STAT3; thus, the STAT3 is phosphorylated via the JAK, and this activation is preferred by the PKM2, which leads to the promotion of serine hydroxymethyltransferase 2 transcription in cancer [44,45]. The dimerized form of the PKM2 and the STAT3 enhances the glycolytic effect, which leads to enhanced exosome secretion. On the contrary, the phosphorylated PKM2 performs as a protein kinase, thus phosphorylating the SNAP23 and consequently promoting the SNARE complex formation, and hence activating the exosome release [39,43]. TDEs could play a pivotal role in the induction of cancer cachexia [38].

Exosomes may also transfer small 19–24 nucleotide-long, non-coding RNAs called microRNAs (miRNAs) that regulate gene expression [46,47,48,49,50,51,52]. Lin et al., found that exosome-derived miR-3184-5p expression levels were lowered in gastric cancer (GC) patients. miR-3184-5p has been shown to bind X-box binding protein 1 (XBP-1), a potential transcription activator of STAT3 that plays a critical role in cancer transformation and carcinogenesis [25]. Thus, downregulated miR-3184-5p may inhibit gastric cancer cell proliferation (Figure 2). However, silencing of XBP-1 was also shown to decrease the expression of p65, p-AKT, and p-STAT3, thereby inducing cell apoptosis (Table 1) [23,24,25]. TDEs advance pro-tumor inflammation via toll-like receptor (TLR) signaling (Figure 2). In lung cancer, the tumor cell-secreted exosomal miR-21 and miR-29a showed that they could connect to the TLR-8, inducing the activation of the NF-kB that leads to the production of pro-inflammatory cytokines, metastasis, and tumor proliferation (Figure 2) [2]. The study by De Veirman et al., found that multiple myeloma cell-derived exosomes may deliver miR-146a, which may affect not only the JAK/STAT pathway but also the Notch (via targeting Numb, a repressor of the Notch signaling pathway) and MAPK pathways (Table 1) [3]. Numerous studies suggest a crosstalk between JAK/STAT and Notch signaling, with each pathway capable of suppressing the other (Table 1) [3,20,22]. In addition to the above-mentioned role of tumor-derived exosomes, HPV infection generates cervical cancer cells that secrete CXCL10 and CXCR3 complexes binding to fibroblasts and activating the JAK/STAT pathway, which leads to the upregulation of the exosomal programmed death-ligand 1 (PD-L1), creating resistance to cell death (Figure 2) and forming an immune escape response pathway that leads to tumor emergence [53].

## 3. Potential Therapeutic Implication of JAK/STAT Pathway

### 3.1. Signal Transducer and Activator of Transcription Factors (STATs)

STATs are regulating tumor proliferation, differentiation, and apoptosis [55]. STATs consist of 750–900 amino acids and have two phosphorylation sites: one of them is tyrosine 705, which is necessary for the STAT activation, and the other one is serine 727, which is phosphorylated after the tyrosine and does not lead to transactivation by itself. These are essential for STAT dimerization and transactivation. The activation of STAT begins with the binding of extracellular signals such as epidermal growth factors (EGF) and cytokines, which leads to the dimerization of the receptors [56,57,58].

#### 3.1.1. STAT1

STAT1 can be activated by cytokines, IFNs, IL-2, IL-6, different growth factors, tumor necrosis factor (TNF), and angiotensin II. STAT1 has numerous functions like regulation of cell cycle-related genes or inhibition of cyclin expression. These lead to the inhibition of cell growth [59,60]. STAT1 may also regulate cell differentiation through phosphorylation and promote apoptosis by expressing multiple apoptosis proteins [61].

In osteosarcoma tissues, collagen type VI alpha 1 (COL6A1) is highly expressed [62,63]. It has been revealed in other cancer types as well, like prostate cancer, renal cell carcinoma, and cervical cancer, and these are associated with poor survival outcomes [64]. Cancer-associated fibroblasts activate fibroblasts that reside in the TME and produce a large number of chemokines, growth factors like TGF-β, cytokines like IL-1β, IL-6, IL-8, and collagen [65]. COL6A1 can be packed into exosomes and internalized by cancer cells, while its overexpression was shown to improve cell motility and metastasis in pancreatic cancer [66]. STAT1 significantly increases cell cycle arrest and apoptosis and decreases the capability of colony formation, cell migration, and cell invasion by suppressing epithelial-mesenchymal transition in osteosarcoma cells with COL6A1 overexpression [67].

#### 3.1.2. STAT2

STAT2 is mostly activated by type I IFNs, especially FN-α and IFN-β [68]. STAT2 is different from other members of the family, as it is the only STAT that is not capable of forming homopolymers and does not directly bind to DNA [69]. Its functions are the antiviral effects via interferon-stimulated genes and the regulation of immune responses [68]. The transcription factor STAT3 is initiated by IFN-I, IL-6, and IL-10 family members, along with IL-21, IL-27, G-CSF, and leptin [70,71,72,73]. It is the main negative immune regulator of cell growth, differentiation, apoptosis, immune response, and tumor metastasis, and is also responsible for signal transduction processes [74].

#### 3.1.3. STAT3

The JAK/STAT3 pathway has a key role in the growth and development of many human cancers. Elevated levels of IL-6 are frequently observed in malignancies and stimulate hyperactivation of JAK/STAT3 signaling (by induction of VEGF, cyclin D1, and MMPs), often associated with poor patient outcomes [75]. STAT3 activation influences the expression of the vascular endothelial growth factor (VEGF) and the hypoxia-inducible factor 1-alpha (HIF1-α), which leads to angiogenesis (Figure 2) [63]. Moreover, exosome surface-expressed Hsp72 in myeloid-derived suppressor cells (MDSCs) also showed the ability to trigger STAT3 in a TLR-2/MyD88-dependent manner leading to autocrine IL-6 production [40].

Chen et al., found that hBM-MSCs promote GC growth by regulating *c*-*Myc*. In addition to GC development being regulated by *c*-*Myc*, it also takes place in breast cancer cell proliferation as it suppresses apoptosis via upregulating the genes *cyclin D1* and *c-MYC* with the help of the STAT3 pathway [76]. The higher level of expression of the *cyclin D1* increases the loss of expression of *MSH2*, which leads to the appearance of Lynch syndrome. The increased expression of the *c*-*Myc* influences the expression of the exosomal human telomerase reverse transcriptase (hTERT). The exosomal hTERT-mRNA level is more elevated in Lynch syndrome mutation carriers (MSH2, MSH6), especially in metastatic colon cancer [77]. Exosomes, with the help of STAT pathways, could be a novel intermediate in the part of MSCs in GC promotion, and MSC-derived exosomes may be an unexplored therapeutic target not only for GC and ovarian cancer treatment but also for novel diagnostics of Lynch syndrome [46,76,78,79,80].

Ham et al., found that glycoprotein 130 (gp130) found in TDEs activates the IL-6/STAT3 pathway in macrophages, thereby helping to form the tumor microenvironment [31,81]. Gp130 is a signal transduction receptor for a cytokine family, which is involved in JAK/STAT signaling [31,72,82]. TDEs can transfer gp130 onto the bone marrow-derived macrophage cell membrane, where it can further accumulate. The inhibition of exosomal gp130 may reverse the effects of TDEs on macrophages [31]. TDEs can transfer gp130 onto the bone marrow-derived macrophages cell membrane where it can accumulate. For this inhibition, the implementation of EDTA is a viable approach [9]. TDEs pre-treated with SC144 lower the exosome-activated STAT3 level and additionally decrease the bone marrow-derived macrophages’ nuclear translocalization level. The incubation of the MDMs with pre-treated TDEs changes the IL-6 secretion phenotype [83]. Likewise, pre-treated TDEs mediate a decrease in gp130/STAT3 gene expression. In addition, in the bone marrow-derived macrophages, TDE-generated morphological and pro-survival changes were reverted by the pre-treated TDEs. All results confirm that exosomal gp130 plays a pivotal role in the STAT3 activation and changes in IL-6 secretion, as well as in the enhancement of survival and morphological changes in bone marrow-derived macrophags reacting to TDEs [31,83]. The study by Ying et al., found that ovarian cancer cells may release exosomes that carry miR-222-3p to macrophages. miR-222-3p overexpression may induce the polarization of the M2 phenotype in macrophages (Table 1) [27]. This type produces IL-10 at high levels, thereby promoting cancer cell proliferation. This regulation is initiated by the STAT3 pathway [84]. Also, inhibition via exosomes of the post-KRAS shows decreased levels of miR-210 expression, the downstream target of which is STAT3 in lung- and pancreatic-related tumors [29,30,85,86]. Inhibiting the STAT3 pathway might have therapeutic potential in ovarian cancer, as this pathway plays a pivotal role in the migration, invasiveness, and proliferation of tumor cells [27,87].

Recent studies have demonstrated that exosomes generated by senescent neutrophils, which are commonly present in therapy-treated tissues, can induce a resistant phenotype in recipient cancer cells. Exosomal piRNA-17560 secreted by senescent neutrophils through the STAT3-dependent pathway plays a crucial role in inducing chemoresistance and promoting epithelial-mesenchymal transition in breast cancer cells. These insights offer a potential approach to enhance therapeutic effectiveness by leveraging exosomes derived from senescent neutrophils [88].

#### 3.1.4. STAT4

STAT4 is activated by type I IFN, IL-12, and IL-23 [89]. STAT4 phosphorylation is needed for the humoral immune response; it activates the germinal center response when viruses invade the body [90]. It was demonstrated that miR-141 can target the STAT4 gene expression to inhibit the proliferation, migration, and invasion of liver cancer cells [91]. On the other hand, the pro-oncogenic role of miR-141/200c in mature T-cell lymphoma cells via the altered expression of genes regulating cell survival and differentiation, including STAT4, has been shown. Moreover, an association between miR-141/200c-driven downregulation of STAT4 with an immature phenotype and shortened survival in primary T-prolymphocytic leukemia cases has been demonstrated [92]. STAT4 is also a target of miR-155, and inhibition of this oncogenic miRNA leads to the upregulation of STAT4 in MyLa cells [93]. In such cases, using engineered exosomes to deliver miRNA mimics or miRNA sponges may serve as a potential therapeutic strategy.

#### 3.1.5. STAT5

STAT5 is mainly activated by the IL-2 cytokine family and IL-3 prolactin, but it can also be activated by the plate-derived growth factors EGF, EPO, GM-CSF, TPO, and GH. STAT5′s main biological function is the regulation of tumor immunity, development, cell growth, differentiation, and apoptosis [94,95,96,97,98]. High doses of renal cancer-derived exosomes inhibit STAT5 phosphorylation in T lymphocytes. TDEs are also known to suppress natural killer (NK) cells’ recruitment, migration, proliferation, and survival by reducing phosphorylation of STAT5 [99,100]. There are two major therapeutic approaches to counter such effects: the removal of circulating TDEs or blocking their formation and release [101,102] and abolishing the adverse effects of TDEs on NK cells using IL-15 [103,104]. Apart from restoring the expression of NK group 2D receptors, IL-15 may also restore STAT5 phosphorylation levels in NK cells [105]. Ye et al., found that in nasopharyngeal carcinoma, which is an Epstein–Barr virus-associated malignancy, the increased concentration of exosomes is in tune with a developed lymphoid node stage and poor prognosis [106]. TW03-derived exosomes inhibit T-cell proliferation along with the differentiation of the Th1 and Th17 [106,107]. Moreover, they advance Treg induction via NPC cells [108]. The above-mentioned outcomes are associated with a drop in ERK, STAT1, and STAT3 phosphorylation and the advancement of the STAT5 phosphorylation in the exo-stimulated T-cells. Also, TW03-derived exosomes enrich the proinflammatory cytokines such as IL-1β, IL-6, and IL-10 but reduce the IL-2, and IL-17 and IFNγ release from CD4+ and CD8+ T-cells. In nasopharyngeal carcinoma, there are overexpressed miRNAs in the exosomes, which were isolated from patient serum and cells, namely, hsa-miR-24-3p, hsa-miR-891a, hsa-miR-106a-5p, hsa-miR-20a-5p, and hsa-miR-1908 [106].

#### 3.1.6. STAT6

IL-4 and viruses may activate STAT6. The functions of STAT6 are to promote B cell proliferation and maturation and to mediate the expression of MHC II and IgE [109]. A novel approach in immunotherapy is checkpoint inhibition. In metastatic cancer, this method induces a long-lasting tumor response [110,111]. The most promising checkpoint immunotherapy is reprogramming immunosuppressive tumor-associated macrophages (TAMs) to generate antitumor immunity. However, within the tumor microenvironment, myeloid cells are the primary antagonist mechanism to checkpoint immunotherapy [112]. TAMs are myeloid subgroups in the TME that show an immunosuppressive phenotype like the M2 [110,113,114]. Clinical data indicate a strong association between TAMs and poor prognosis. The M2 phenotype is regulated by STAT6, which has an impact on the TAMs. Upon interleukin stimulation, activated STAT6 dimerizes and alters the nucleus, which influences the transcription of M2 signature genes, in addition to suppressing the activation of M1 or inflammatory genes [115,116,117]. A novel approach is exosome-based TAMs reprogramming. Others have also investigated this method which selectively distributes STAT6 targeting antisense oligonucleotides (ASO) to TAMs [118]. exoASO-STAT6, in the wake of intravenous administration in the liver, exhibits biodistribution and STAT6-silencing activity with minimal distribution to other tissues. exoASO-STAT6 displays potent antitumor activity in numerous preclinical tumor models by inducing remodeling of the tumor microenvironment. exoASO-STAT6 applied via intravenous or intratumoral in syngenic tumor models appertaining to colorectal cancer and hepatocellular carcinoma proceeded to significant inhibition of tumor growth and complete tumor remission [110,119].

To investigate the effect of non-small cell lung cancer cells on the differentiation of the M2 macrophages, MRC-5 cells, and H1299 cell lines were co-incubated with PMA-stimulated THP-1 cells [120]. The results showed an intensified M2 macrophage number in the H1299 cell culture medium [121]. GW4869 inhibits M2 and macrophage marker secretion, suggesting that in H1299 cells the differentiation of the M2 macrophage happens through exosome secretion [122]. Also, they found that the exosomes from H1299 cells are more homogeneous and more spherical than typical exosomes, with a diameter of around 99 nm [123]. H1299-derived exosomes diminished the level of M1 markers and raised the expression in the M2 markers. On the other hand, the pretreatment with an exosome inhibitor showed no impact on M1 and M2 markers [123]. Other studies have revealed that macrophage-specific target gene transcription is activated by STAT6, which results in the differentiation of the M2 macrophage [124,125]. LINC00313 is linked to the expression of the STAT6. M1 markers like INOS, CD86, TNF-α, and IL-1β have increased expression levels if LINC00313 is knocked down, along with reduced M2 markers like TGFβ expression, which is caused by the overexpression of the STAT6. The expression of the CD206 along with the CD163-positive M2 cells also decreases if the LINC00313 is knocked down, whereas it increases when the STAT6 is overexpressed. These results indicate that the LINC00313 expression has an impact on the differentiation of the M2 macrophages via the STAT6 upregulation [120].

### 3.2. Janus Kinase Signaling Pathways

There are four members of the JAK family: JAK1, JAK2, JAK3, and tyrosine kinase 2. These are non-receptor tyrosine protein kinases. JAK1 may be phosphorylated by cytokine subunits, such as γc receptors, class II receptors, and gp130 subunits [126]. JAK1 may phosphorylate all the members of the STAT family [127]. It is expressed in all tissues and may induce hematopoiesis after activation via IL-3, IL-5, and IL-7 [128]. Mesenchymal stroma/stem-like cells (MSCs) are known for their antitumor activity. MSC-derived exosomes induce tumor cell growth, metastasis, and invasion. Glycoprotein A repetition predominant (GARP) is an oncogene in breast cancer. Deregulated GARP has been reported to promote aggressive tumor biology. siGARP-MSC exosomes inhibit the secretion of IL-6 and also inactivate the JAK1/STAT3 axis [129,130,131,132,133]. As such exosomes also reduced the proliferation and invasion of a mouse colon cancer cell line, a group of authors have proposed them as a potential novel anti-cancer tool worth investigating in the human context [133].

#### 3.2.1. JAK1

The JAK/STAT3 pathway is one of the modulators of the MSC’s immunosuppression [134]. Phosgen is a highly poisonous gas and an intermediate product in different types of medicines, plastics, and dyes [135,136]. Exposure to phosgens could cause acute lung injury and cancer. To this day, there is no effective treatment to reverse the damage induced by phosgen [137]. Lung-derived exosomes upregulate the expression of the miR-28-5p, which promotes the MSCs function in phosgen induce acute lung injury. On the other hand, lung-derived exosomes reduce the manifestation of the miR-34c-3p, which activates the MSCs function via the JAK1/STAT3 pathway [138]. Suppressors of cytokine signaling (SOCS) are intracellular proteins that negatively regulate the JAK/STAT pathway as well as inflammation and immune responses [139]. Several studies have targeted the inhibition or activation of the JAK/STAT pathway’s different movements. Masoumi-Dehghi et al., found that the ovarian tumor cell-derived small extracellular vesicles upregulate the JAK-STAT pathway via down-regulating the SOCS5 [140,141]. Notably, the inhibitory effect of these extracellular vesicles on the SOCS5 expression level was partially rescued when transfected with anti-miR-141. It is indicated that JAK1 and STAT3 transcript expression levels were dramatically enhanced in TD-sEV-stimulated HUVECs; however, the stimulating impact of TD-sEVs on JAK1 and STAT3 expression levels was somewhat inhibited in the presence of anti-miR-141-3p. Also, the reduced SOCS5 expression resulting from small extracellular vesicles transfer of tumor-secreted miR-141-3p upregulates the expression of VEGFR-2 by stimulating the JAK/STAT signaling pathway in endothelial cells [142]. The ovarian tumor cell-derived small extracellular vesicles upregulate the VEGFR-2, thereby promoting endothelial cell migration and angiogenesis in vitro [139,143].

#### 3.2.2. JAK2

JAK2 is also phosphorylated by the cytokine subunit, gp130 receptor family, and the class II cytokine receptor family. It is a signal transductor of receptors of IL-3, growth hormone, and prolactin [144]. Zhang et al., found that renal cancer-derived exosomes downregulated the expression of JAK3 but there was no effect on the expression of JAK2 in T-lymphocytes (Figure 2) [35]. Exosomes and tumor-associated macrophages have complex crosstalk in the glioma microenvironment [145]. Ming et al., studied the glioma progression and the macrophage polarization correlation with the effect of the TDE [146]. Firstly, they looked for functional miRNAs that can only be found in glioma by sequencing the cerebrospinal fluid and glioma tissues. miR-3591-3p met the criteria perfectly; it can also significantly influence the M2 macrophage polarization and the secretion of the IL10 and TGFB1, which leads to glioma metastasis, and its overexpression induces apoptosis [147]. It can also activate different pathways like the JAK2 pathway and STAT3 pathway. Lentivirally transduced macrophages via miR-3591-3p significantly stimulate glioma progression. This study revealed that tumor-suppressive miR-3591-3p is secreted by exosomes in glioma cells and targets tumor-associated macrophages to generate an immunosuppressive microenvironment [146,148,149]. The next step was to examine the expression level of the miR-3591-3p in exosomes by examining it in different glioma and normal astrocyte cell lines. Increased expression level of exosomal miR-3591-3p was found in the studied glioma cell lines. Investigating the influence of glioma cells on macrophages in the tumor microenvironment, they co-cultured glioma cells THP-1-Mφ yielded with PMA in vitro. In these groups, they gauged the M1 and M2 macrophage-associated phenotypic marker levels via qRT-PCR. The level of M2 markers was significantly increased in the glioma co-cultured groups, on the other hand, the exosome release inhibitor-treated group’s M1 markers decreased [146].

Chim et al., studied SOCS1 and SHP1 hypermethylation in multiple myeloma. SOCS1 may bind to the JAK2 kinase SH2 domain, inhibiting its activity, and is also a negative regulator of IL-6; therefore, it may function as a tumor suppressor in multiple myeloma. SHP1 is also a negative regulator of the JAK/STAT signaling pathway. The studied genes were shown to be inactive due to methylation; therefore, demethylating agents may be potential therapeutic agents [21].

#### 3.2.3. JAK3

JAK3 expression is only limited to hematopoietic progenitor cells and is mainly required for γc receptor signal transduction. JAK3 deregulation may lead to combined autosomal recessive immunodeficiency [150,151]. Differential microarray analysis showed that in prostate cancer tissue, the miR-187 expression decreased, which is correlated with poor prognosis in prostate cancer patients. On the other hand, prostate cancer cells’ malignant phenotype is inhibited by miR-187 overexpression [26]. The JAK3/STAT3-slug axis, which is reduced by human bone marrow-derived mesenchymal stem cell-derived exosomes miR-187, maintains prostate cancer metastasis and cell growth (Table 1) [26]. In multiple myeloma cells, metastasis can advance by CD276 via activation of the JAK/STAT pathway [152]. Overexpressed CD276 has been reported in numerous malignant tumors, which were reported to advance the metastasis of cancer cells. Overexpressed CD278 correlates with poor clinical prognosis [26,152].

## 4. Conclusions

This review has shown the promising roles of exosomes in diagnostic and therapeutic applications. These vesicles provide a potential tool for assessing tumor progression and malignant state; however, they may also be engineered to complement antitumor therapy. Several strategies to modify the tumor microenvironment have been suggested, including the use of TDE membrane-anchored interleukins and TDE-encapsulated miRNAs affecting the JAK/STAT signaling pathway. Exosome processing and analysis were not addressed in this review, but since the workflow has not been standardized yet, the methodology varies between studies, which may result in inconsistent findings. Nevertheless, exosomes and TDE modifications show clear promise for personalized diagnostics and therapy.

## Data Availability

Not applicable.
